# Dysregulation of miR-21-5p, miR-93-5p, miR-200c-3p and miR-205-5p in Oral Squamous Cell Carcinoma: A Potential Biomarkers Panel?

**DOI:** 10.3390/cimb44040121

**Published:** 2022-04-16

**Authors:** Ovidiu Aghiorghiesei, Oana Zanoaga, Lajos Raduly, Alexandra Iulia Aghiorghiesei, Paul Chiroi, Andrada Trif, Rares Buiga, Liviuta Budisan, Ondine Lucaciu, Laura Ancuta Pop, Cornelia Braicu, Radu Campian, Ioana Berindan-Neagoe

**Affiliations:** 1 Department of Oral Health, “Iuliu Haţieganu” University of Medicine and Pharmacy, 15 Victor Babeș Street, 400012 Cluj-Napoca, Cluj, Romania; aghiorghiesei.ovidiu@umfcluj.ro (O.A.); patricia.lucaciu@umfcluj.ro (O.L.); 2 Research Center for Functional Genomics, Biomedicine and Translational Medicine, “Iuliu Haţieganu” University of Medicine and Pharmacy, 15 Victor Babeș Street, 400012 Cluj-Napoca, Cluj, Romania; oana.zanoaga@umfcluj.ro (O.Z.); lajos.raduly@umfcluj.ro (L.R.); chiroi.paul@umfcluj.ro (P.C.); liviuta.petrisor@umfcluj.ro (L.B.); ioana.neagoe@umfcluj.ro (I.B.-N.); 3 Department of Prosthetics and Dental Materials, “Iuliu Haţieganu” University of Medicine and Pharmacy, 15 Victor Babeș Street, 400012 Cluj-Napoca, Cluj, Romania; irimie.alexandra@umfcluj.ro; 4 Emergency County Hospital, 3-5 Clinicilor Street, 400000 Cluj-Napoca, Cluj, Romania; andrada_ramona_trif@yahoo.com; 5 Department of Pathology, The Oncology Institute “Prof. Dr. Ion Chiricuta”, 34-36 Republicii Street, 400015 Cluj-Napoca, Cluj, Romania; rares.buiga@yahoo.fr; 6 Department of Pathology, “Iuliu Hatieganu” University of Medicine and Pharmacy, 15 Victor Babeș Street, 400012 Cluj-Napoca, Cluj, Romania; 7 Department of Oral Rehabilitation, “Iuliu Haţieganu” University of Medicine and Pharmacy, 15 Victor Babeș Street, 400012 Cluj-Napoca, Cluj, Romania; rcampian@umfcluj.ro

**Keywords:** oral cancer, squamous cell carcinoma, miRNAs and lncRNA profiling, immunohistochemistry

## Abstract

Oral squamous cell carcinoma (OSCC) is considered the sixth most common cancer worldwide. To reduce the high mortality of the disease, sensitive and specific diagnostic and prognostic biomarkers are urgently needed. Non-coding RNA, microRNAs (miRNAs), which are short length non-coding transcripts, or long non-coding RNA (lncRNA) seem to be potential biomarkers, considering that they have an important role in regulation of cell fate being involved in a wide range of biological processes. Literature data emphasized the important role of these transcripts as a biomarker for diagnosis and prognosis in oral squamous cell carcinoma. Therefore, we have evaluated the expression levels of a panel of four miRNAs (*miR-21-5p*, *miR-93-5p*, *miR-200c-3p* and *miR-205-5p*) and *H19*, *MALAT1* by quantitative real-time PCR (qRT-PCR) from 33 fresh frozen tissues and 33 normal adjacent tissues. Our date revealed *miR-21-5p* and *miR-93-5p* to be upregulated, while *miR-200c-3p* and *miR-205-5p* to be downregulated. Regarding the long non-coding RNAs, *H19* and *MALAT1*, were also downregulated. We also investigated the expression of *BCL2*, which is another important gene correlated to non-coding RNAs investigated by as, and it was also under-expressed. Additional validation step at protein level was done for KI67, TP53 and BCL2. In our patient cohort no correlation with clinical stage and smoking status was observed. The results of the present study indicated the important role of *miR-21-5p*, *miR-93-5p*, *miR-200c-3p*, *miR-205-5p* and *H19* in OSCC. Differential expression of these transcripts at sub-sites, may serve as a diagnostic marker with further elaboration on a larger sample size. Additional studies should be conducted to confirm the results, particularly the interconnection with coding and non-coding genes.

## 1. Introduction

Oral squamous cell carcinoma (OSCC) is considered the sixth most common cancer worldwide (over 200,000 newly diagnosed cases annually), revealing an increasing mortality and incidence according GLOBOCAN 2020 [1]. OSCC accounts for 90–95% of oral malignancies and is principal located in three sites: buccal mucosal, tongue, and lip [2]. Sundermann et al. observed that the oral tumors are localized broadly including sites like mouth, jaw, tongue, buccal cavity, maxilla, hard palate and other parts of the oral cavity [3].

Despite the advancement in diagnostic and management protocols, as well as the prevalent accessibility of prognostic markers there is no improvement of the 5-year survival rate for this cancer type, which ranges from 45 to 50% [2,4,5]. The occurrence of OSCC is a complex multistep process, being represented by an overlap between the genetic background, microenvironment imbalance and the presence of adverse factors promoting carcinogenesis. Among these factors, betel quid chewing, cigarette smoking, and alcohol drinking habits are the main causes of the high incidence of OSCC [2]. Moreover, variations in risk factors and associated clinical parameters across different world geographical areas add more complexity and tumor heterogeneity to OSCC [6].

Increasing evidence show that multiple coding and non-coding genes are involved in OSCC. The discovery of new cancer molecular targets can also efficiently help to comprehend the pathogenesis and prognosis of OSCC and develop new therapeutic strategies [7,8]. Accumulating documentation proved that non-coding RNAs (ncRNAs), such as microRNAs (miRNAs), act a as crucial regulatory element in cellular physiological/pathological processes [9,10]. miRNAs are transcripts of 19–25 nucleotides long and without known protein-coding potential [11], they may act as oncogenes or tumor suppressors and be potential indicators for prognosis in various tumors, including OSCC [12]. One of the main challenge in evaluating the clinical role of the noncoding genes, is except few miRNAs that are consistently expressed in almost all cancers, the majority have different expression level due to tumor heterogeneity, stage, gender, associated TME and other criteria [13]. In this light, aberrant expression of some miRNAs had been shown to be closely correlated with OSCC prognosis and diagnosis, the expression levels being correlated with clinical characteristics [11]. While suffering from the same diagnostic patients with OSCC have different localization that can also alter the tumors’ profiling [14,15].

Long non-coding RNAs (lncRNAs) are transcripts with over 200 nucleotides (nts) and without known protein-coding potential [11], they may also act as oncogenes or tumor suppressors and could be used as potential biomarkers for different hallmarks of cancer including OSCC [12]. Aberrant expression of some lncRNAs has been shown to be closely correlated with OSCC prognosis and diagnosis, the expression levels being corelated with clinical characteristics [9]. A well-known long noncoding RNA, *H19*, has both tumor promoter and suppressive roles, those being cell type or site specific [4,16].

This could be a reason why in the literature many miRNAs and lncRNAs are found altered in OSCC both upregulated and downregulated, results being consistent only based on the patients’ cohorts investigated, their demographic and clinical data.

Dysregulation of *miR-21-5p, miR-93,* are closely connected to the development of different cancers. *miR-205* and *miR-200* family of miRNAs play divergent function in different cancer types [17]. The role of these miRNAs in cancers related mechanisms remain largely uncovered. In the present study, we have evaluated the expression levels of these four miRNAs: *miR-21-5p*, *miR-93-5p*, *miR-200c-3p* and *miR-205-5p* and *H19*, *MALAT1*, long non-coding RNA, and *BCL2* by quantitative real-time PCR (qRT-PCR) in 33 fresh frozen OSCC tumors and their adjacent normal tissue samples. The expression of BCL2, TP53 and KI67 was also evaluate using immunohistochemistry (IHC) for gene expression validation and to better understand the correlation of miRNAs, long non-coding RNAs and genes in oral cancer. The evaluation was done considering the association of tested targets expression levels with tumor characteristics and smoking status, to elucidate the underlying signaling pathways using in silico tools.

## 2. Materials and Methods

### 2.1. Patients

This study included a cohort of 33 patients who were diagnosed with oral squamous cell carcinoma. For the 33 patients we had fresh frozen tumors and their paired adjacent normal tissue. The diagnosis was established using internationally accepted criteria. More details on the clinical data of the study population are presented in Table 1. Informed consent was obtained from all subjects involved in the study. 

### 2.2. RNA Extraction

Fresh frozen tissue was used for RNA extraction using the classical Phenol-Chloroform method. Mainly the tissue was homogenized in 800 µL TripleXtractor (Grisp, Porto, Portugal) and then the sample was used for RNA extraction. First the sample was treated with chloroform (160 µL), mixed well by vortex, incubated at room temperature (RT) for 5 min and centrifuge 20 min at 13,000× *g* rpm and 4 °C. The transparent phase was transferred to a new 1.5 mL tube and RNA was precipitated with 500 µL of isopropanol, mixed by tube inversion and incubated 15 min at RT, then centrifuged at 13,000× *g* rpm and 4 °C for 15 min. The supernatant was removed, and the pellet was washed with 1ml of 75% ethanol and centrifuged 5 min at 10,000 rpm and 4 °C. After removal of the ethanol, the pellet was left to air-dry for 10–15 min, and then dissolved in 25 µL nuclease free water. The obtained RNA was quantified using NanoDrop (ThermoFischer Scientific, Waltham, MA USA) spectrophotometer. 

### 2.3. Evaluation of miRNAs by Real Time Quantitative PCR

For the evaluation of expression of *miR-21-5p*, *miR-93-5p*, *miR-200c-3p* and *miR-205-5p*, the obtained RNA was reverse transcribed using the TaqMan MicroRNA Transcription kit (ThermoFischer Scientific, Waltham, MA USA) and TaqMan microRNA primer assay (ThermoFischer Scientific, Waltham, MA USA) for the selected miRNAs and *U6* and *RNU48* as housekeeping miRNAs. Therefore, 1 µL of total RNA was mixed with 0.75 µL of 10× RT Buffer, 0.1 µL of RNase inhibitor, 0.075 µL dNTP, 0.1825 µL of each of the 20× miRNA RT primers, 4.52 µL of nuclease free water and 0.5 µL of MultiScribed RT enzyme. The mixture was incubated at: 16 °C for 30 min, 42 °C for 30 min, 85 °C for 5min and hold at 4 °C. The cDNA obtained was diluted six times with nuclease free water and then used in the real time qPCR reaction. We made a mixture of 5.03 µL of ready to use TaqMan Fast Advance Master Mix (ThermoFischer Scientific, Waltham, MA USA) and 0.47 µL of TaqMan microRNA primer and added 5.2 µL of cDNA for each of the miRNA analyzed. From this mixture we added 5 µL to two wells of the PCR plate. The PCR program used in the Viia7 instrument was as follows: 1 cycle-2 min at 50 °C, 1 cycle-20 s at 95 °C and 40 cycles at 95 °C-1 s and 60 °C-20 s in the FastMode. The obtained C_T_ values were analyzed using the ΔΔC_T_ method and the obtained results were imported in GraphPad Prism software v.9 (GraphPad Software, San Diego, CA, USA) for further analysis. In Table 2 are presented the sequence of the primers of the tested miRNAs.

### 2.4. Evaluation of Long Non-Coding RNAs and Gene Expressions by Real Time Quantitative PCR

For the evaluation of the expression level of *H19* and *MALAT1* lncRNAs and *BCL2* gene, the obtained RNA was reverse transcribed using the High-Capacity cDNA Reverse Transcription Kit (ThermoFischer Scientific, Waltham, MA USA). To the RNA sample we added 2 µL of 10× RT buffer, 0.8 µL of 25× dNTP, 2 µL of 10× Random Primer, 1 µL of Multiscribe Reverse Transcriptase, 0.25 µL RNase inhibitor and 3.95 µL of nuclease free water. This mixture is incubated: 10 min at 25 °C, 120 min at 37 °C, 5 min at 85 °C and hold at 4 °C. After cDNA synthesis the obtained cDNA is diluted 1:5 with nuclease free water. For gene expression analysis we used the ready to used SYBR Select Master Mix (ThermoFischer Scientific, Waltham, MA USA) and 2.5 µL diluted cDNA. We used *B2M* and *GAPDH* as housekeeping genes. Over the cDNA we added 10 µL of Sybr Select Master Mix, 0.1 µL of primer 100 µM or 0.2 µL of primers 50 µM and 8.8/8.6 µL of nuclease free water. This mixture was split into two wells of the PCR plate, 10 µL in each well. The PCR program used in the ViiA7 instrument was as follows: 1 cycle-2 min at 50 °C, 1 cycle -2 min at 95 °C and 40 cycles at 95 °C-15 s and 60 °C -30 s. The obtained C_T_ values were analyzed using the ΔΔC_T_ method and the obtained results were imported in GraphPad Prism software v.9 (GraphPad Software, San Diego, CA, USA) for further analysis. In Table 3 and Table 4 are presented the sequence of the primers of the tested lncRNAs and gene.

### 2.5. Evaluation by Immunohistochemistry of KI67, TP53 and BCL2

Expression of Ki-67, TP53 and BCL2 proteins was done using a standard immunohistochemical protocol. From each case, the most representative tumor block was selected, coming from the tumor biopsy performed at the time of the anatomopathological diagnosis, before therapy. Tumor tissues fixed in 10% buffered formalin and embedded in paraffin, according to the classical histological method, were sectioned at 5 μm and stained automatically using a Roche Ventana Benchmark Ultra automatic staining device. The following primary antibodies were used: for Ki67: anti-Ki-67 (30-9) Rabbit Monoclonal Primary, ready to use, from Ventana (Roche Diagnostics); for BCL2: anti-BCL2 (SP66)-RTU Rabbit Monoclonal Primary antibody, from Ventana (Roche Diagnostics); for TP53: (NCL-L-P53-D07) from Novocastra, recognizing a denaturation resistant epitope between amino acids 1 and 45 in wild type as well as mutant p53 protein. The antibodies were diluted 1:100. Histological sections were incubated with primary antibodies for 1 h, treated with Ultra CC1 for 92 min. OptiView DAB IHC Detection Kit (Roche Diagnostic) was used for staining detection.

In the case of KI67 staining, the tumor proliferation index (PI) was calculated based on the percentage of positive tumor nuclei relative to the total number of epithelial tumor cells present per section. Similarly, the percentage of positive cells for TP53 and BCL2 was calculated.

## 3. Results

### 3.1. qRT-PCR for miRNA Expression Evaluation

**Figure 1:**
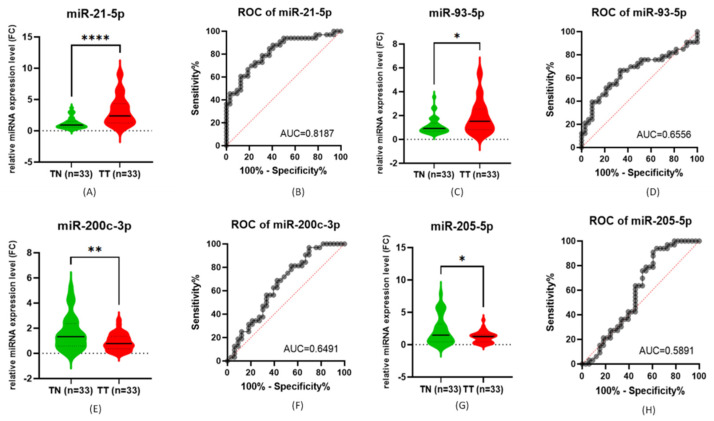
The expression level of tested miRNAs: (**A**) *miR-21-5p*, (**C**) *miR-93-5p*, (**E**) *miR-200c-3p*, and (**G**) *miR-205-5p*; ROC curves of tested miRNAs: (**B**) *miR-21-5p*, (**D**) *miR-93-5p*, (**F**) *miR-200c-3p*, and (**H**) *miR-205-5p* (**** < 0.0001, ** < 0.01 and * < 0.05).

In the analysis of the relative expression level of the tested miRNAs we obtained statistically significant levels for all the tested miRNAs when comparing tumor versus normal adjacent samples. Statistical analysis of each tested miRNAs showed that *miR-21-5p* (26/7, 78.78%) and *miR-93-5p* (20/13, 60.60%) were upregulated, while *miR-200c-3p* (27/6, 81.81%) and *miR-205-5p* (29/4, 87.87%) were downregulated, with a *p* value of <0.0001, 0.01, 0.0079 and 0.0172 (Figure 1). When analyzing the ROC (receiver operating characteristic) curves for the selected miRNAs we observed that *miR-21-5p* had an AUC (Area under the ROC Curve) greater than 0.7 (Figure 1). The AUC for the other miRNAs was lower.

**Figure 2:**
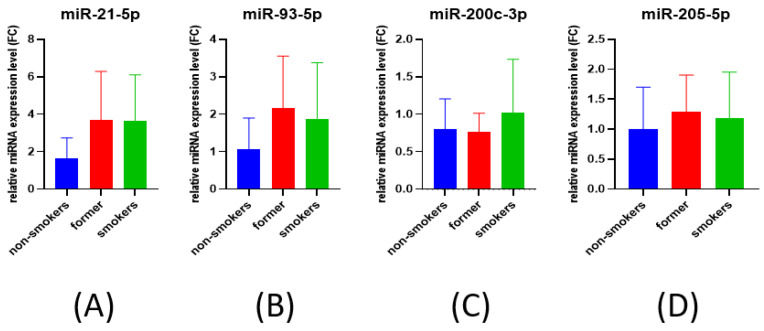
Bar plot for miRNAs expression correlated to smoking status: (**A**) *miR-21-5p*, (**B**) *miR-93-5p*, (**C**) *miR-200c-3p*, and (**D**) *miR-205-5p*.

When analyzing the effect of smoking on miRNAs expression we observed no statistically significant difference between the expressions of smokers, former smokers or non-smokers (Figure 2).

**Figure 3:**
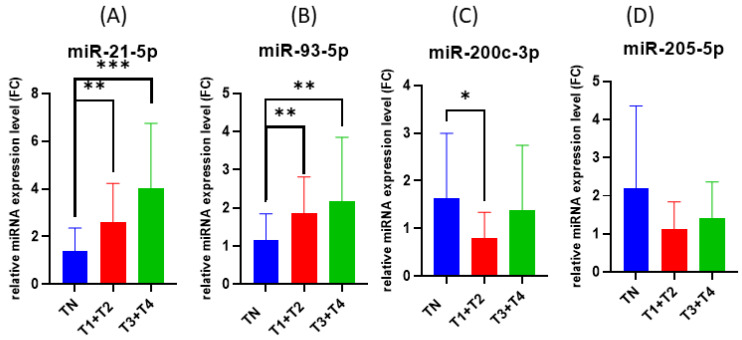
Bar plots comparing miRNAs expression of normal tissue to tumor stage: (**A**) *miR-21-5p*, (**B**) *miR-93-5p*, (**C**) *miR-200c-3p* and (**D**) *miR-205-5p*. *** < 0.001, ** < 0.01 and * < 0.05.

A statistical significant correlation was observed between the expression of miRNAs and tumor stage when compared to normal tissue with respect to *miR-21-5p*, *miR-93-5p* and *miR-200c-3p*, as can be seen in Figure 3.

**Figure 4:**
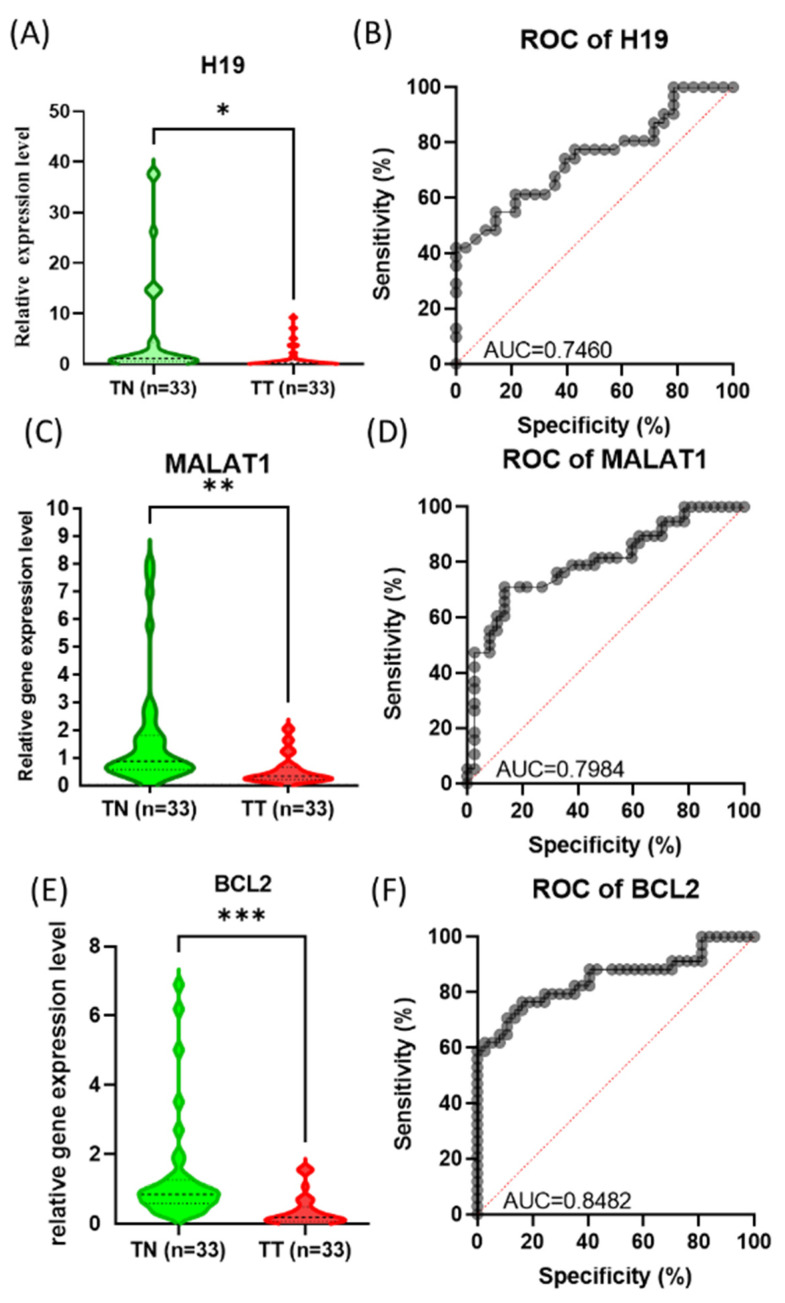
The expression level of (**A**) *H19*, (**B**) *ROC curve for H19*, (**C**) *MALAT1* (**D**) ROC curve for *MALAT1*, (**E**) *BCL2*, and (**F**) ROC curve of BCL2 (*** < 0.001, ** < 0.01 and * < 0.05).

In the analysis of the relative expression level of the tested long non-coding RNA *H19*, *MALAT1* and gene *BCL2* we obtained statistically significant levels when comparing tumor versus normal adjacent samples. *H19*, *MALAT1* and *BCL2* were observed to be underexpressed in the tested samples in comparison to normal samples (Figure 4A,C,E). When analyzing the ROC (receiver operating characteristic) curve for the tested samples, we observed an AUC (Area under the ROC Curve) over 0.7 for all targets, with *BCL2* being the highest (Figure 4B,D,F). As for the case of miRNA expression, the expression of *H19*, *MALAT1* and *BCL2* did not correlate in a statistically significant way with smoking status or tumor stage.

**Figure 5:**
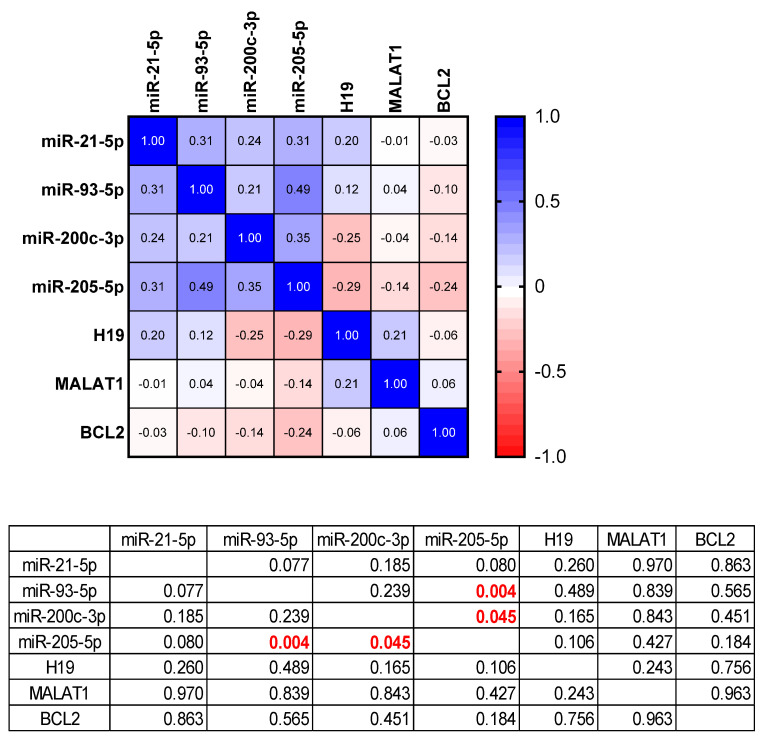
Pearson correlation of the tested targets.

Figure 5 presents the statistical correlation between the expressions of the tested targets, miRNAs, lncRNAs and *BCL2 gene*. The values in red are statistically significant correlations. A direct statistically significant correlation was observed between the expression of *miR-205-5p* with *miR-93-5p* and *miR-200c-3p*. According to the Pearson correlation coefficient for both miRNAs we have moderate positive correlation as can be seen in Figure 5.

### 3.2. Potential Targets for miR-21-5p, miR-93-5p, miR-200c-3p, and miR-205-5p

**Figure 6:**
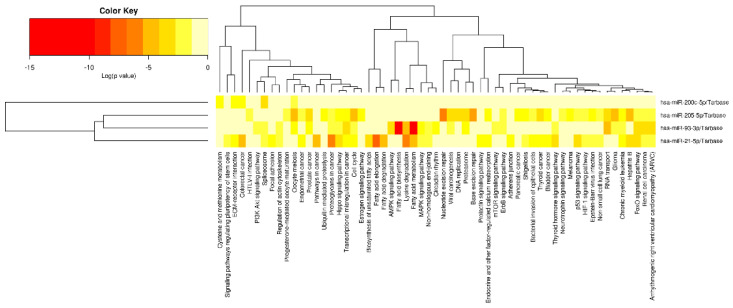
Heatmaps of significant pathways predicted by DIANA-miRPath (www.microrna.gr/miRPathv3, accessed on 10 March 2022) for *miR-21-5p*, *miR-93-5p*, *miR-200c-3p*, and *miR-205-5p.* Pathways are represented on the x-axis and miRNAs on the y-axis. The color code expresses the log (*p*-value), with the most relevant predicted miRNA-pathway interactions are displayed in red.

miRNAs function by regulating the expression of their target genes and therefore the potential targets of the differentially expressed miRNAs were explored. All the miRNAs had experimentally validated miRNA target genes in miRTarBase (Figure 6). 

**Figure 7:**
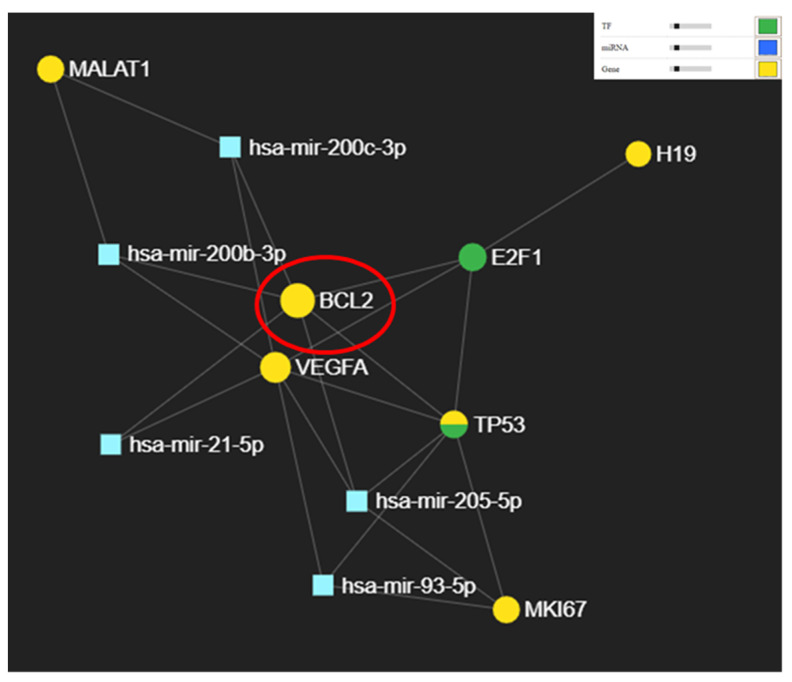
Figure miRNA-lncRNA interaction network generated using miRNET online tool.

Additional miRNA-mRNA-lncRNA network generated using miRNET was done and is presented in Figure 7. *BCL2* is the core of the network being targeted by three of the tested miRNAs. *BCL2* is targeted directly by *miR-21* a transcript having the highest expression level in oral cancer, which is in accordance with the low expression level of *BCL2*.

### 3.3. Evaluation by Immunohistochemistry of KI67, TP53 and BCL2 Gene

**Figure 8:**
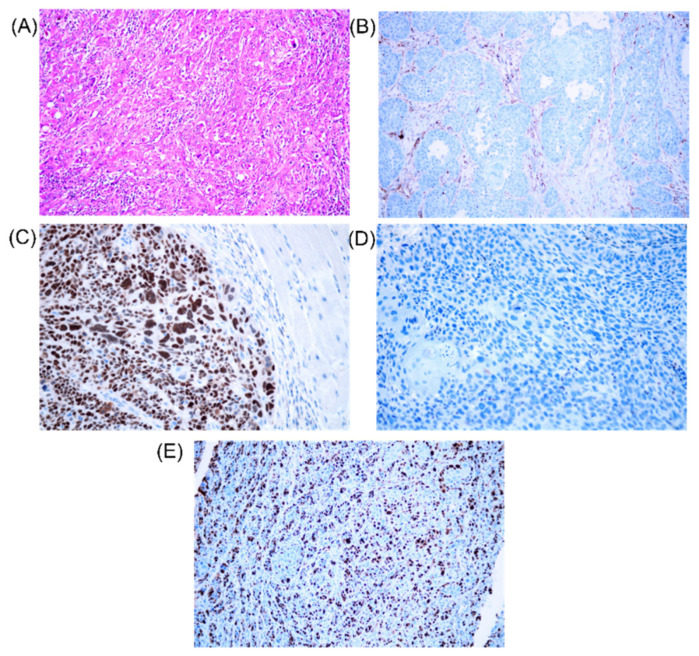
IHC staining squamous carcinoma. (**A**) Squamous cell carcinoma, high grade HE 200×. (**B**) Squamous cell carcinoma, low BCL2 expression, 400× magnification. (**C**) Squamous cell carcinoma, high TP53 expression, 400× magnification. (**D**) Squamous cell carcinoma, low TP53 expression, 400× magnification. (**E**) Squamous cell carcinoma, KI67 high proliferative index, 200× magnification.

Considering the network in Figure 7 we also evaluated the expression of KI67, TP53 and BCL2. KI67 positive cells were observed in most tumors, ranging from 5–60%. In the case of TP53 protein, 15 samples have mutant (mt) TP53: 11 samples <1%, 2 samples: 80%, 1 sample:95%), and 7 wild type (wt) samples having an expression level between 15–65%. Sections from 22 tumors were stained for BCL2 protein, and one of these tumors showed a distinct cytoplasmic staining (90%), 10 of the cases had BCL2 expression ranging from 10–35, and the rest of the cases had low expression levels (0–5%). The individual values are presented in Table 5. Figure 8 presents an example of the IHC slides.

## 4. Discussion

The results obtained in relation to miRNA-signature in the OSCC are similar to other bioinformatics and meta-analysis studies, both in the type of miRNAs (*miR-21-5p*, *miR-93-5p*, *miR-200c-3p*, and *miR-205-5p*) and in their expression (up or downregulated) [6,18,19,20,21,22,23]. A difference in the percentage of deregulated miRNAs (up or downregulated) was observed in the function of the affected areas [14,24]. In the case of the mouth, the floor of mouth, tongue, and base of tongue samples revealed a higher number of downregulated miRNAs than upregulated, whereas lip and tonsil samples presented the opposite [25]. 

Accumulating data revealed an overexpression of *miR-21* in several solid tumors [26], a recent meta-analysis demonstrated the oncogenic role of *miR-21* in HNSCC [27] or OSCC [28]. We must emphasize that *miR-21* has been proposed as a prognostic marker on numerous occasions in OSCC [19,23,24], this was highlighted in our qRT-PCR study. In a recent study among the 64 microRNAs, *miR-21-5p* exhibited the best statistical performance (area under the curve = 0.972; 95% confidence interval: 0.911–1.000) in differentiating between OSCC tumor tissue and healthy mucosa [29]. Another study presented *miR-21* as a metastasis promoter in OSCC, and the high expression levels for this transcript were significantly correlated with the lymph node classification [23]. Additionally, it was observed that *miR-21-5p* had the greatest accuracy in differentiating between superficial and deep tumors [30]. Other study revealed *miR-21* expression was significantly expressed at the different OSCC subsites; the highest expression level was observed in buccal mucosa, following the gingival, buccal and tongue OSCC, study of Pryia et al. [24].

Overexpression of *miR-93-5p* was related to increase migration and invasion characteristics in squamous cell carcinoma of the head and neck [15,31], revealing oncogenic role, but the mechanisms of action still needs to be deciphered [31]. Additionally, its high expression was correlated to EMT and was associated with response to radiotherapy and poor prognosis [22,31,32]. A meta-analysis study showed that *miR-21* and *miR-93-5p* are up regulated in oral cancer [33,34].

The *miR-200* family is a group of tumor suppressor miRNAs involved in the regulation of metastatic potential of tumor cells [35], by regulation of the epithelial-to mesenchymal transition, repression of self-renewal and differentiation of cancer stem cells and chemoresistance [36]. The correlation between *miR-200* family dysregulation and cancer prognosis remains controversial [37]. Previous studies have shown inconsistent data related to *miR-200c-3p* [18,36]. A study done on OSCC (*n* = 40) and normal (*n* = 8) tissues demonstrated that *miR-200a*, *miR-200b*, *miR-200c* and *miR-141* are significantly downregulated in OSCC [36]. Downregulation of the *miR-200* family miRNAs was related with tobacco chewing/smoking risk habits and undifferentiated pathology [36]. *miR-200c-3p* promote invasive capacity in OSCC microenvironment, being overexpressed in exosomes’ fraction in the OSCC patients versus healthy controls [18].

A study done on 71 primary OSCC specimens and 28 metastatic lymph node OSCC samples, observed that *miR-205-5p* is down-regulated in the primary OSCC tissue samples than in normal adjacent oral mucosa tissue samples, and its expression is lower in the metastatic lymph node tissue samples than in the primary OSCC tissue samples [38].

When analyzing the correlation of miRNAs expression to genes we observed an interconnection with a key gene involved in apoptosis, *BCL2* and angiogenesis *VEGFA*, Figure 7. Interconnection with key lncRNA retrieved altered in solid tumors like *HOTAIR, H19, XIST* or *MALAT1*, among this we selected for validation on our patient cohort *H19* and *MALAT1* considering the reduced number of studies for this non-coding transcripts available. Additional interconnection with the validated proteins by IHC (KI67 alias MKI67, BCL2 and TP53) can be observed in the same figure. Additionally, we should consider to investigate the functional relationship between miRNA, lncRNA, and mRNA on genome-wide scale for better understanding the regulatory roles of this transcripts in OSCC biology.

In our study, we found a significant downregulation of *MALAT1* in OSCC tumors compared to adjacent normal tissue. Only few cancer types (breast cancer, esophageal carcinoma, sarcoma, thymoma and uveal melanoma) have a reduced expression level of *MALAT1* [4]. The TCGA analysis displayed increased expression of *MALAT1* in the HNSCC tumors and it was also related with gene amplification in 4% of HNSCC patients [4]. The BCL2 expression in IHC studies has been correlated to the gene expression observed in the RT-PCR experiment, being low in most of the studied cases. The same was observed in a study on 30 oral cancer patients by Arya et al. [39].

The expression levels for *MALAT1* and *H19* was downregulated in tumor tissues of 62.5% and 81.25%, respectively, of OSCC patients [4]. In same study, it was observed that *MALAT1* and *H19* negatively correlated with the smoking status of patients [4]. *H19* is linked with diverse cancer types and has both tumor promoter and suppressive roles [4]. *H19* was downregulated in tumor tissues of 84.3% of OSCC patients, which is in accordance with our TCGA dataset analysis on HNSCC tumors compared to normal tissue [4].

## 5. Conclusions

In conclusion, the results of the present study revealed upregulation of *miR-21-5p* and *miR-93-5p*, and downregulation of *miR-200c-3p* and *miR-205-5p*. Additionally, no correlation between dysregulated expression of the tested miRNAs and lncRNAs and tobacco/smoking status in OSCC was obtained.

The identification of alteration of *miR-21-5p*, *miR-93-5p, miR-200c-3p*, *miR-205-5p*, *H19* and *MALAT1* could be a useful target for clinically application in OSCC. Additional validation as molecular biomarkers for early diagnosis, prognostic monitoring and appropriate therapy in larger patient cohort should be considered. Once more, the heterogeneity of the OSCC due to different localization is influenced by numerous tissular factor, environmental ones and habits aspects like smoking status. Differential expression of this transcripts at sub-sites, may serve as a diagnostic marker with further elaboration on a larger sample size.

## Figures and Tables

**Table 1:** Clinical data for all the patients included in the study.

	Fresh Frozen Tissue	
Characteristics	Cases (*n* = 33)	Percentage %
**Age**		
≤60	11	33.33
>61	22	66.67
NA	0	0.00
**Sex**		
Females	7	21.21
Males	26	78.79
**LNM status**		
Negative	16	48.48
Positive	14	42.42
NA	3	9.09
**TNM status**		
T1	8	24.24
T2	6	18.18
T3	6	18.18
T4	11	33.33
NA	2	6.06
**Smoking**		
Yes	24	72.73
No	5	15.15
Former	3	9.09
NA	1	3.03
**Differentiation**		
Well	11	33.33
moderate	18	54.55
Poor	3	9.09
NA	1	3.03
**Location**		
Head	3	9.09
Tongue	6	18.18
Tonsils	1	3.03
Lip	5	15.15
Mandible/maxilla	6	18.18
Oral cavity	9	27.27
Gum	1	3.03
Palate	2	6.06

**Table 2:** Sequences of tested miRNAs.

miRNA	Assay Code	Mature Sequence
RNU48	0001006	GATGACCCCAGGTAACTCTGAGTGTGTCGCTGATGCCATCACCGCAGCGCTCTGACC
U6	001973	GTGCTCGCTTCGGCAGCACATATACTAAAATTGGAACGATACAGAGAAGATTAGCATGGCCCCTGCGCAAGGATGACACGCAAATTCGTGAAGCGTTCCATATTTT
miR-21-5p	000397	UAGCUUAUCAGACUGAUGUUGA
miR-93-5p	001090	CAAAGUGCUGUUCGUGCAGGUAG
miR-200c-3p	002300	UAAUACUGCCGGGUAAUGAUGGA
miR-205-5p	000509	UCCUUCAUUCCACCGGAGUCUG

**Table 3:** Primer sequence for *H19*, *MALAT1*, *BCL2*, *GAPDH* and *B2M*.

Gene	Primer Sequence
B2M	F:TTACTTCCTCCACGGAGTCG
	R:TGAGCTGGGTAGCACCATTT
GAPDH	F:ATCTTCCAGGAGCGAGATCCC
	R: TGAGTCCTTCCAAGATACCAA
H19	F:CACCCCCACTGAAAAAGATGAG
	R:CCTCCATGATGCTGCTTACATG
MALAT1	F:TGTCCTTATAGGCTGGCCATT
	R:AACTGCAGAGTTTGAGTGGTTTT

**Table 4:** Primer sequence for BCL2.

Gene	Primer Sequence
BCL2	F:AGTACCTGAACCGGCACC
	R:GCCGTACAGTTCCACAAAGG

**Table 5:** Summary of immunohistochemical expression of KI67, TP53 and BCL2 proteins (“mt”—mutant proteine expression; “wt”—wild type protein expression).

No Sample	PI (KI67)	TP53%	Bcl2%
1	30	<1 (mt)	0
2	5	<1 (mt)	5
3	25	<1 (mt)	35
4	15	<1 (mt)	10
5	25	<1 (mt)	0
6	35	80 (mt)	0
7	15	<1 (mt)	10
8	25	60 (wt)	20
9	5	80 (mt)	0
10	50	<1 (mt)	30
11	60	<1 (mt)	10
12	55	<1 (mt)	15
13	40	65 (wt)	5
14	60	90 (mt)	5
15	40	<1 (mt)	5
16	10	25 (wt)	15
17	35	95 (mt)	1
18	25	25 (wt)	0
19	20	<1 (mt)	5
20	7	15 (wt)	0
21	10	15 (wt)	15
22	15	60 (wt)	20

## Data Availability

Not applicable.
